# COVID-19 Detection Based on Image Regrouping and Resnet-SVM Using Chest X-Ray Images

**DOI:** 10.1109/ACCESS.2021.3086229

**Published:** 2021-06-04

**Authors:** Changjian Zhou, Jia Song, Sihan Zhou, Zhiyao Zhang, Jinge Xing

**Affiliations:** Key Laboratory of Agricultural Microbiology of Heilongjiang ProvinceNortheast Agricultural University 12430 Harbin 150030 China; Department of Modern Educational TechnologyNortheast Agricultural University 12430 Harbin 150030 China; College of Electrical and InformationNortheast Agricultural University 12430 Harbin 150030 China

**Keywords:** COVID-19, medical image processing, deep learning, Resnet-SVM

## Abstract

As the COVID-19 spread worldwide, countries around the world are actively taking measures to fight against the epidemic. To prevent the spread of it, a high sensitivity and efficient method for COVID-19 detection is necessary. By analyzing the COVID-19 chest X-ray images, a combination method of image regrouping and ResNet-SVM was proposed in this study. The lung region was segmented from the original chest X-ray images and divided into small pieces, and then the small pieces of lung region were regrouped into a regular image randomly. Furthermore the regrouped images were fed into the deep residual encoder block for feature extraction. Finally the extracted features were as input into support vector machine for recognition. The visual attention was introduced in the novel method, which paid more attention to the features of COVID-19 without the interference of shapes, rib and other related noises. The experimental results showed that the proposed method achieved 93% accuracy without large number of training data, outperformed the existing COVID-19 detection models.

## I. Introduction

Corona virus disease 2019 (COVID-19) has spread over the world, resulting in more than 156 million confirmed cases and 3.2 million deaths in 192 countries or regions till early May, 2021, especially in India there were even more than 400 thousands new confirmed cases in a single day [1]. Due to its long incubation period (14 days) and no obvious early symptoms, make it easily missed the best treatment period, together with its highly contagious nature, which lead to a large-scale outbreak of the epidemic. Early diagnosis of COVID-19 becomes an urgent task to prevent spread of the disease. The Reverse Transcription Polymerase Chain Reaction (TR-PCR) is a widely used method for COVID-19 detection, which detects viral nucleic acid to confirm whether the tester is infected. However, this method may give a low accuracy by using nasopharyngeal and throat swabs, which can be affected by low viral load and sampling errors [2].

Since the chest computed tomography (CT) screening on initial patient presentation had showed more outperforming sensitivity and more accuracy than RT-PCR. However, the increasing patients with suspected COVID-19 made the limited CT places overburdened even more. Therefore, the chest X-ray (CXR) for recognizing COVID-19 features is more and more needed. Unfortunately, the initial chest X-ray findings have a lower accuracy than that of RT-PCR [3].

With the accumulation of staff experience, the sensitivity and accuracy of CXR based COVID-19 detection had increased a high level, which had been a useful tool for detecting COVID-19 cases. Especially the machine learning based auxiliary diagnostic technology had been used widely, more and more researchers had been focus on it.

According to the published papers, the COVID-19 detection area is mainly focused on the traditional machine learning algorithm based raw chest X-ray images classification, Hussain *et al.* [4] proposed an AI imaging analysis tool to classify COVID-19 lung infection based on portable CXR images. The authors introduced five supervised machine learning models for COVID-19 CXR images classification, the classifiers such as XGB-L, XGB-Tree, CART (DT), KNN, and Naïve Bayes were used in the work. Experimental result showed that the combined classifier had achieved 100% accuracy on two class classification and 79.52% on multi-class classification. Zimmerman and Kalra [5] gave a review of several cardiovascular applications of machine learning models and their potential applications to cardiovascular diagnosis and therapy in COVID-19 infection. The authors analyzed the COVID-19 early detection and risk stratification tools and found that the machine learning algorithms can give a software solution application with the ability to analyze large amounts of training data and make predictions without prior programming. When faced with new problems with unique challenges as evident in the COVID-19 pandemic, the machine learning algorithms can offer solutions quickly through massive quantities of training data and making associations that may have been missed.

As the deep convolution neural network method had achieved an unprecedented advantage in image processing, for COVID-19 detection, there are large numbers of achievements had been achieving. Oh *et al.* [6] proposed a patch-based CNN method using a relatively small number of trainable parameters for COVID-19 diagnosis; the authors were inspired by their statistical analysis of the CXR radiographs potential imaging biomarkers, and designed a deep neural network with limited training samples for COVID-19 CXR images analysis. To this purpose, the authors first investigated the CXR images analysis imaging biomarkers and proposed a random patch cropping based network for classification. Experimental results showed that the proposed method can clearly reflects the radiological findings. Ozturk *et al.* [7] proposed an automatic raw chest X-ray images based COVID-19 detection method, the authors used the DarkNet and YOLO for classifying the COVID-19 images into binary classes (COVID-19 or not) and multi classes (COVID-19, Not, Pneumonia). The method was developed by 17 convolutional layers and introduced different filtering on each layer and can be employed to assist radiologists in validating their initial screening. The proposed method had achieved the 98.08% binary classes’ accuracy and 87.02% multi classes’ accuracy. Karhan and Akal [8] proposed a Chest X-Ray image analysis and classification model based on ResNet-50. First, the authors analyzed the COVID-19 images and identified the infected individuals of the chest X-ray images, then input them into ResNet-50 for classification. The experimental results were encouraging in terms of the use of computer-aided in the field of pathology. Yan *et al.* [9] established a new deep CNN tailored for segmenting the chest CT images with COVID-19 infections, the authors were inspired by the boundary of the infected lung can be enhanced by adjusting the global intensity, they introduced a feature variation block which adaptively adjusts the global properties of the features for segmenting COVID-19 infection.

Arias-Londoño *et al.* [10] presented an evaluation of different methods based on a deep CNN automatic COVID-19 diagnosis tool using chest X-Ray images. The aim of the authors was to evaluate how preprocessing the data affects the results and improves it’s explain ability, and the critical analysis was also analyzed in this system, the accuracy of proposed method had achieved 91.5% and an 87.4% average recall for the worst.

Liu *et al.* [11] proposed a two-dimensional sparse matrix profile dense network method to detect COVID-19 by chest CT images, the authors introduced a matrix profile technique to detect COVID-19 in two levels. First, the CT images were simply flatted and transformed to a one-dimensional and directly classified, and second, a matrix profile was calculated in a sliding window way for every segment in two dimensional. From this method, the severity score and the sparse anomaly mask were calculated to penalize the pixel values of each CT image; finally the VGG-19 model was used for comparison. Compared to the original images, the anomaly weighted images had showed generally better performance in training the DenseNet, which was validated using COVID-19 lung CT image datasets. Rajaraman *et al.* [12] proposed a COVID-19 detection approach by using deep iteratively pruned model, the authors introduced a custom convolutional neural network and the best performing models are iteratively pruned to reduce complexity and improve efficiency, the different ensemble strategies were combined to improve classification performance, experimental results showed that the best-performing pruned models had achieved the 99.01% accuracy and area under the curve of 0.9972 in detecting COVID-19 findings on CXRs.

There were numbers of researchers had used multi-classifier fusion algorithms and transfer learning methods to classify COVID-19 lung infection using CXR images. Mohammed *et al.* [13] proposed an end-to-end weakly-supervised COVID-19 detection method, which named ResNet+, and the novelty approach can provide slice level prediction only require volume level labels. The spatial features were extracted in this approach incorporated a lung segmentation mask and the LSTM was utilized to acquire the axial dependency of the slices, finally, before the final fully connected layer a slice attention module was applied to generate the slice level prediction, experimental results showed that the novelty approach can achieve 81.9 accuracy and 81.4% F1 score, which demonstrated the effectiveness of it. De Moura *et al.* [14] proposed a novel automatic approach specifically tailored for the classification of chest X-Ray images into normal, pathological and COVID-19, and used a 3-complementary method based dense network, the joint method can enhance the identification ability that manifest characteristics similar to COVID-19 and normal cases. Experimental results showed that the proposed approaches had provided a global accuracy of 79.62%, 90.27% and 79.86% respectively. Horry *et al.* [15] proposed a novel method for COVID-19 detection by transfer learning using X-Ray, Ultrasound, and CT scan images. The authors identified a suitable CNN model after comparative study of several popular models, and selected VGG-19 for image modalities and proposed a pre-processing stage to create a COVID-19 dataset for developing and testing. The proposed approach was aimed to reduce the unwanted noise from the images so that deep learning models can focus on detecting diseases with specific features from X-Ray images. Experimental results showed that with the limited training data, most of the deeper networks struggled to train well and provided less consistency. Hilmizen *et al.* [16] proposed a combined two different transfer learning models method for CT-Scan and CXR images classification. The authors collected 2,500 CT images and 2,500 CXR images into two classes of normal and COVID-19, in this work, they used Densenet-121, Mobile net, X-ception, Inception-v3, ResNet-50 and VGG-16 for classification. Experimental result showed that the ResNet-50 and VGG-16 had achieved the highest accuracy of 99.87%. The proposed method was confirmed a better performance than the method of single modality of biomarkers.

Furthermore, as the limited training data for COVID-19 detection research, many scholars began to carry out relevant research work from the data level to improve the classification capacity of models. Tabik, *et al.* [17] proposed a COVID smart data based Network (COVID-SDNet) for improving the generalization capacity of classification models. The authors had demystified the achieved of recent COVID-19 classification models and built a homogeneous and balanced includes all levels of severity database named COVIDGR-1.0, contains 426 positive samples and 426 negative ones in CXR views. The proposed approach can help early detection COVID-19 with the severity level labels. Peng *et al.* [18] presented a public dataset called COVID-19-CT-CXR, which was consist of COVID-19 CXR and CT images, these images were automatically extracted from COVID-19-relevant articles from the PMC-OA Subset. In order to demonstrate the utility of the proposed database, the authors used them as additional training data, was able to contribute to improved deep learning performances for the classification of COVID-19 and non-COVID-19 CT, and the authors designed an unsupervised one-class classifier from non-COVID-19 CXR and performed anomaly detection to detect COVID-19 CXR, this work was complementary to existing resources. Waheed, *et al.* [19] presented a method named Covid-GAN which could generate synthetic chest X-ray (CXR) images auxiliary classifier generative adversarial network based model, this method was proved that the synthetic images generated from the proposed method can enhance the performance of CNN for COVID-19 detection, without Covid-GAN the classification by CNN alone yielded 85% accuracy while the accuracy increased to 95% after adding synthetic images generated by the proposed method, which will lead to more robust systems of radiology.

Jiang *et al.* [20] proposed a CT image synthesis approach based on a conditional GAN that can generate high-quality and realistic COVID-19 CT images for use in deep learning based medical imaging classification tasks. Experimental results showed that after used the proposed method, it outperformed other state-of-the-art image synthesis methods with the generated COVID-19 CT images and indicated promising for various machine learning applications including classification, semantic segmentation and so on.

**FIGURE 1.**
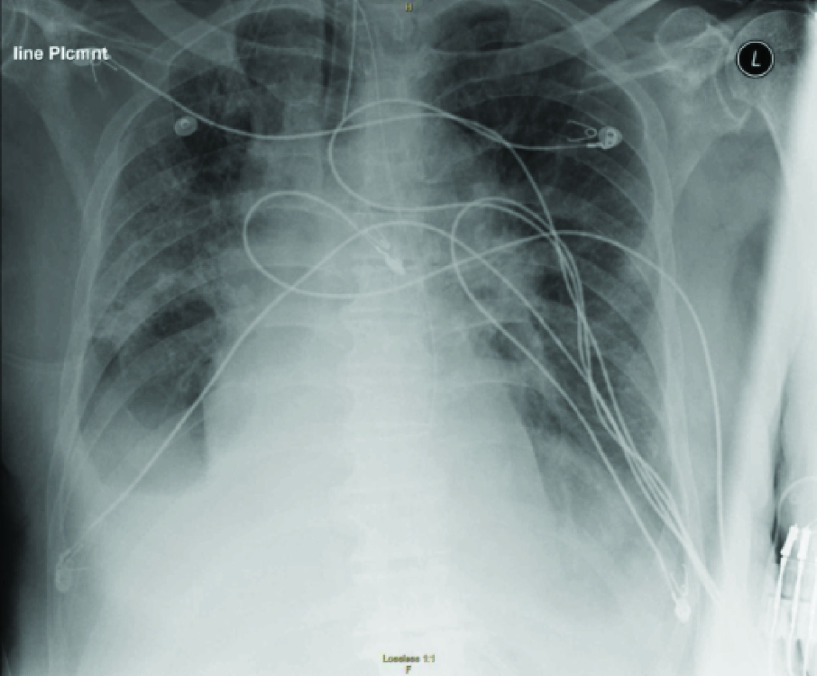
Original CXR image.

All of the mentioned methods above had achieved satisfactory performance. However, the original CXR images contains a large number of background noise, as shown in FIGURE 1, except lung there are lines, shoulder, skeleton and so on, these information can largely interfere the sensitivity of classifiers, although there are some models had achieved the state-of-art performance, the background features were most likely used for feature learning. The training data with a large number of background noises may obtain perfect results, but in practice, these noises will cause great interference to the recognition effect.

In this study, we aimed to propose a novel method which only focuses on the features of lung in CXR images, the proposed method first masked with the lung masks from the segmentation networks, and then further divided the lung area into small rectangular blocks and regrouped them into a square block. In addition, we had come up a new feature extraction model that can extract image features automatically based on deep residual network and Support Vector Machine (SVM) [21]. The main contributions of this study are as follows.

• An image regrouping and ResNet-SVM based COVID-19 detection model was proposed.
• The proposed model can achieve a satisfactory result while not need large-scale training data.
• The visual attention has been successfully applied in this study.

The rest of the paper is organized as follows: Section II presents the related works. The proposed method is discussed in Section III. Experimental and analysis are discussed in Section IV. The conclusion is presented in Section V.

## II. Related Works

The segmentation network, deep learning models and SVM were used in the proposed method in this study. As a contrast, we had trained several state-of-art models, such as AlexNet [22], ResNet-50 [23], DenseNet-121 [24], VGG-16 [25] and so on. In feature extraction stage, the Principal Component Analysis (PCA) [26] method was used for comparison.

### A. Segmentation Network

**FIGURE 2.**
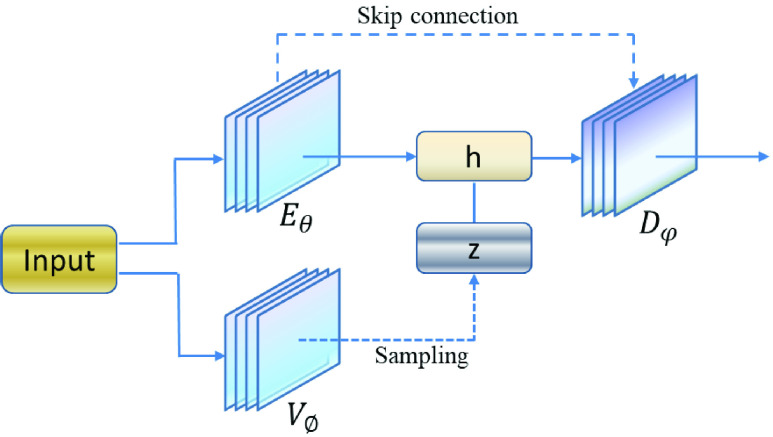
VAE method for segmentation.

In this study, the variational data imputation method for lung segmentation from CXR images was adopted [27]. The variational auto-encoder (VAE) in this method was not used as an auto-encoder but as a method to perform cross-domain mapping between the input and the segmentation domains. The method obtained low dimensional representations of the data by introducing the latent random variable and learned a latent representation were used for training models, which can perform data imputation and possibly capture other task specific features. As depicted in FIGURE 2, the input images as the variational encoder $V_{\emptyset } $ output and the segmentation network $\mathrm {E}_{\theta }$ are concatenated to a low dimensional samples and latent space from the latent space. The decoder $D_{\varphi } $ is shared by joint decoder segmentations by the U-net and VAE.

\begin{equation*} L(s,\hat {s})=L_{rec} (s,\hat {s})+KL\left [{ {q_{\emptyset } (z\vert x)\vert \vert p(z)} }\right]\tag{1}\end{equation*}

where $\hat {s} $ is the predicted segmentation, can be come out from (2).

\begin{equation*} \hat {s} =D_{\varphi } \left [{ {E_{\theta } (x)\forall V_{\emptyset } (x)} }\right]=D_{\varphi } \left [{ {h\forall z} }\right]\tag{2}\end{equation*}

where $\forall $ denotes indicate concatenation. $h$ and $z$ are the output of U-net and a sample from the latent space learnt respectively.

### B. Deep Residual Network

Use Let $x_{l} $ denotes the output of $l^{th}$ layer of Convolutional Neural Networks; the non-liner transformation $H_{l} $ can be the output of $(l-1)^{th}$ layer.

\begin{equation*} x_{l} =H_{l} (x_{l-1})\tag{3}\end{equation*}

where $H$ denotes the convolution operation by *ReLU* and dropout [28].

To solve the problem of network degradation when the very deep networks, the shortcut connections were introduced that sums the identity mapping between the input layer and output layer, which made it in residual version, the residual block can be directly concatenated when the input and output are of the same dimensions. Then the output $\mathrm {x}_{\mathrm {l}}$ can be demonstrated as

\begin{equation*} x_{l} =H_{l} (x_{l-1})+x_{l-1}\tag{4}\end{equation*}

**FIGURE 3.**
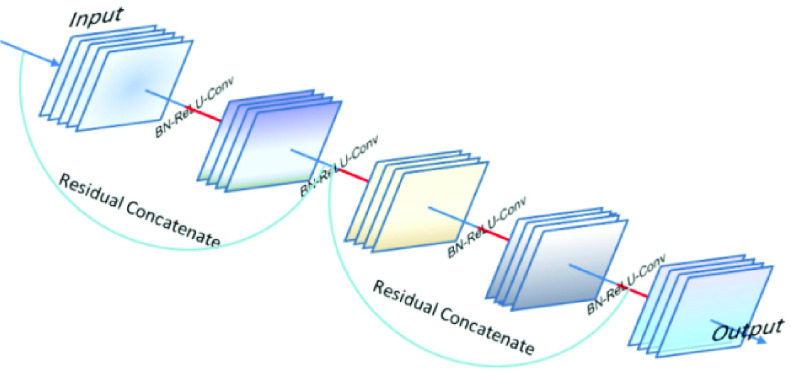
The architecture of deep residual network.

The architecture of deep residual network can be shown in FIGURE 3.

### C. Support Vector Machine

**FIGURE 4.**
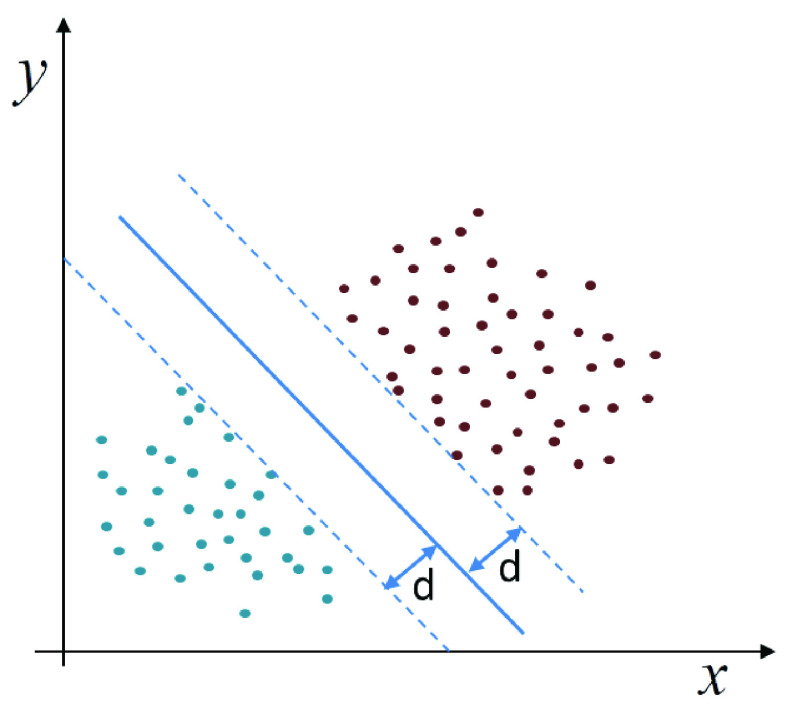
Support vector machine.

As one of the strongest and most powerful machine learning algorithms, Support Vector Machine (SVM) had achieved a large number of praise and application in the period of traditional machine learning [29]. As shown in FIGURE 4, the SVM can find a balance between model complexity and classification ability when given limited sample information. Compared with other machine learning methods, it can overcome the impact of noise and work without any prior knowledge.

The purpose of SVM algorithm is to find a hyperplane which can rationalize the classification effect. All sample points satisfy (5)

\begin{equation*} y\cdot (w^{T}\cdot x_{i} +\gamma)\ge 1\tag{5}\end{equation*}

where $y $ denotes the label of samples, and $x=\left [{ {x_{1},x_{2},x_{3},\cdots x_{n}} }\right]$ denotes the samples, the distance d can be shown in (6).

\begin{equation*} d=\frac {\overline \gamma }{\left \|{ w }\right \|}=\frac {y\cdot (w^{T}\cdot x_{i} +\gamma)}{\left \|{ w }\right \|}=\frac {1}{\left \|{ w }\right \|}\tag{6}\end{equation*}

where $d$ denotes the distance of support vector and the hyperplane. The Lagrangian form was introduced for this optimization:

\begin{equation*} L(w,\gamma,\alpha)=\frac {1}{2}\cdot \left \|{ w }\right \|^{2}-\sum \limits _{1}^{n} {(y_{i} \cdot (w^{T}\cdot x_{i} +\gamma)-1)}\tag{7}\end{equation*}

where the Lagrangian factor $\alpha \ge 0$. Then the optimal solution is (8).

\begin{equation*} \theta (w)=\max \limits _{\alpha _{i} \ge 0} L(w,\gamma,\alpha)=\frac {1}{2}\left \|{ w }\right \|^{2}\tag{8}\end{equation*}

Thus, the purpose of SVM is to calculate the maximum value of $d$ when the value of $\frac {1}{2}\left \|{ w }\right \|^{2}$ is minimum.

## III. Proposed Method

**FIGURE 5.**
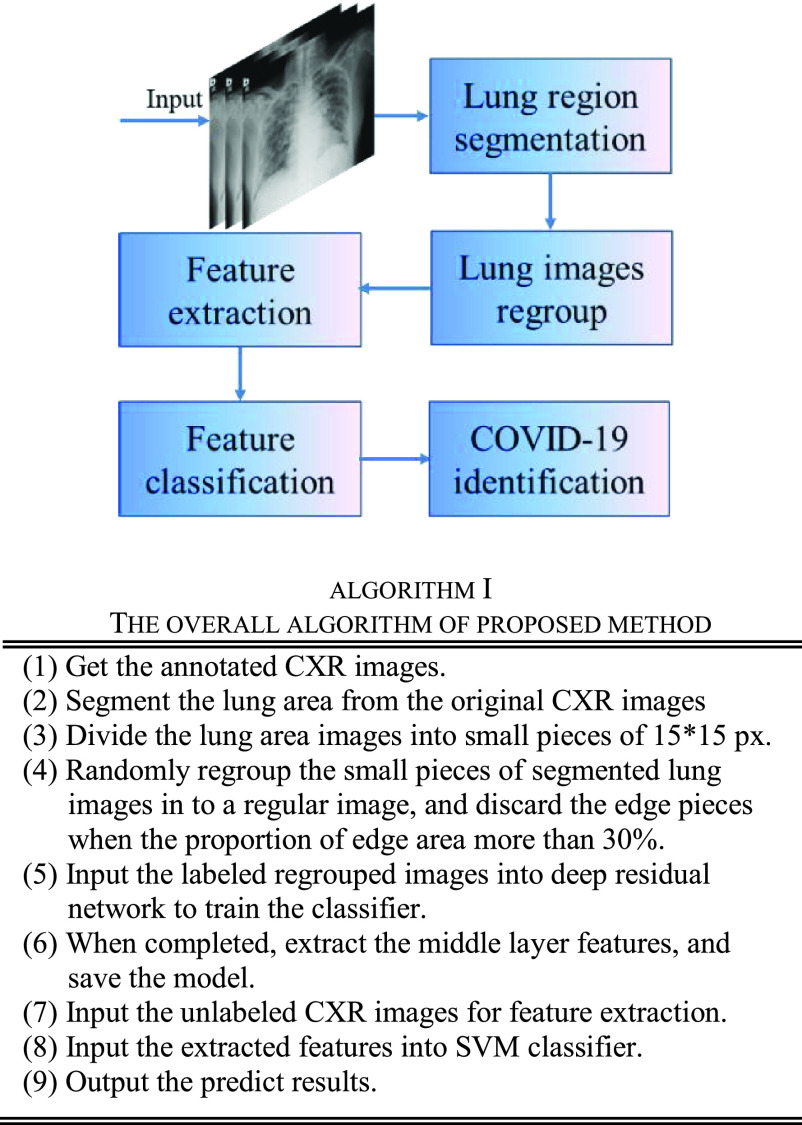
The overall algorithmic framework of proposed method.

The overall algorithmic framework is given in FIGURE 5 and the main stages of the proposed method were described in algorithm I.

### A. Lung Segmentation and Regroup

#### 1) Lung Segmentation

**FIGURE 6.**
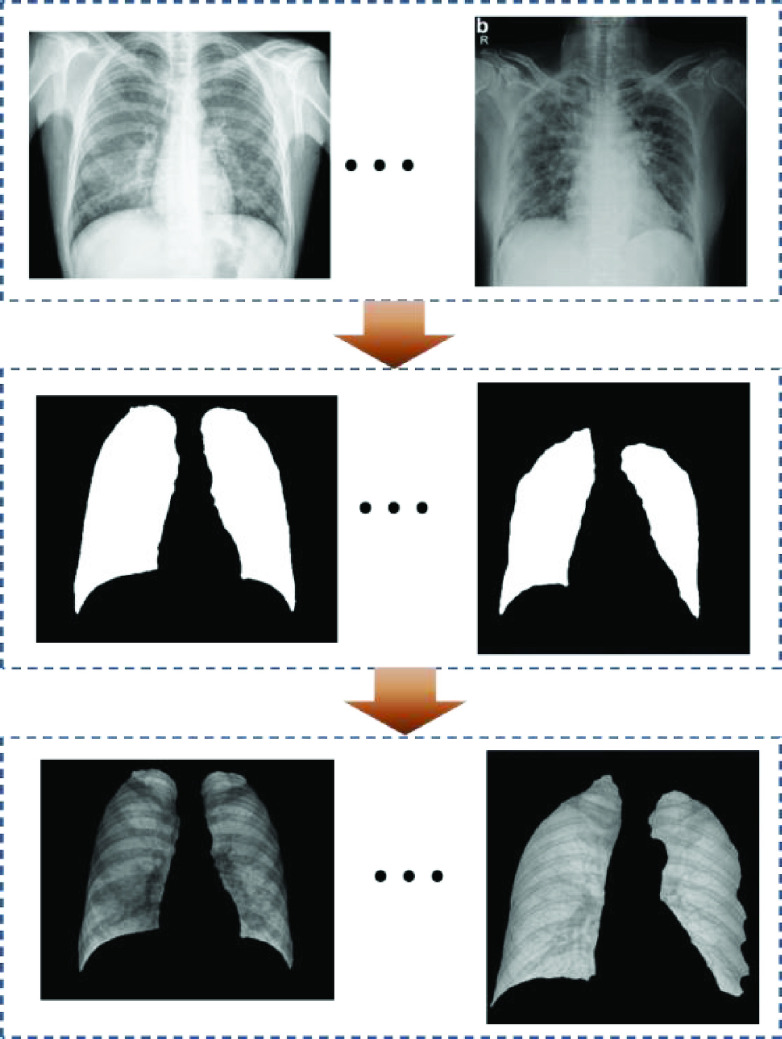
Lung segmentation process.

In this study, the variational data imputation method [27] was used for lung segmentation, the method utilized a liked in the baseline U-net segmentation network, and a data imputation encoder by additional variational. A sequence of 4 1-D convolution layers were used by variational encoder to transform 2-D features maps for predicting and the latent dimension was 8 in this study. The segmented lung images can be seen in FIGURE 6.

#### 2) Lung Images Regroup

**FIGURE 7.**
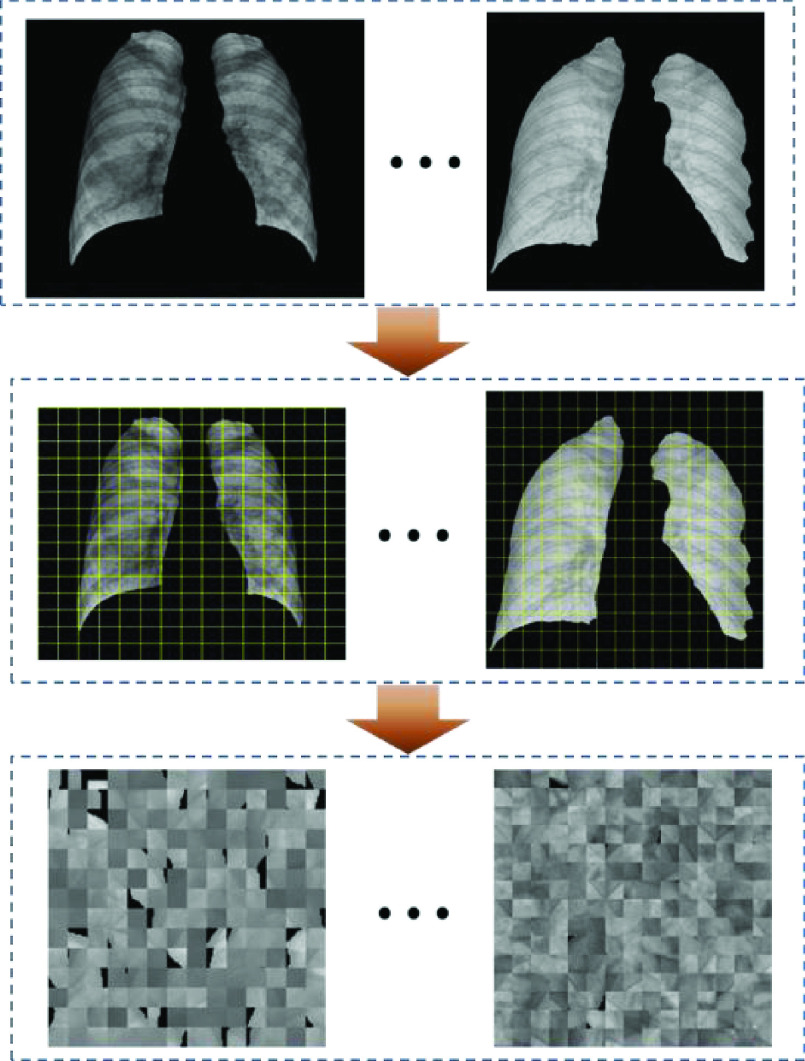
The processes of lung image regroup.

As can be seen from the segmented lung images from FIGURE 6, it contains a lot of background noises and disruptors in lung region such as lines, spine and ribs. These noises have a large interferes with the sensitivity of classifiers. Therefore, it is necessary to get rid of the interference, so that the classifier can focus on COVID-19 detection. Since the lung region has irregular boundaries while the input of classifiers require images to be regular. In this study, we proposed a novel lung image regroup method, which not only discarded the background interference noises, but also weakened the ribs and other interference information. First, the segmented lung images were divided into numbers of $15\times 15px$ pieces, and randomly regrouped them into a regular image. There was a small tips, when the small pieces contains the edge of lung region, there would have a black area in these pieces, in this study, the small pieces should be abandoned when the proportion of black area more than 30%. The process of lung images regroup was depicted in FIGURE 7.

### B. Feature Learning By Extended Deep Residual Network

**FIGURE 8.**
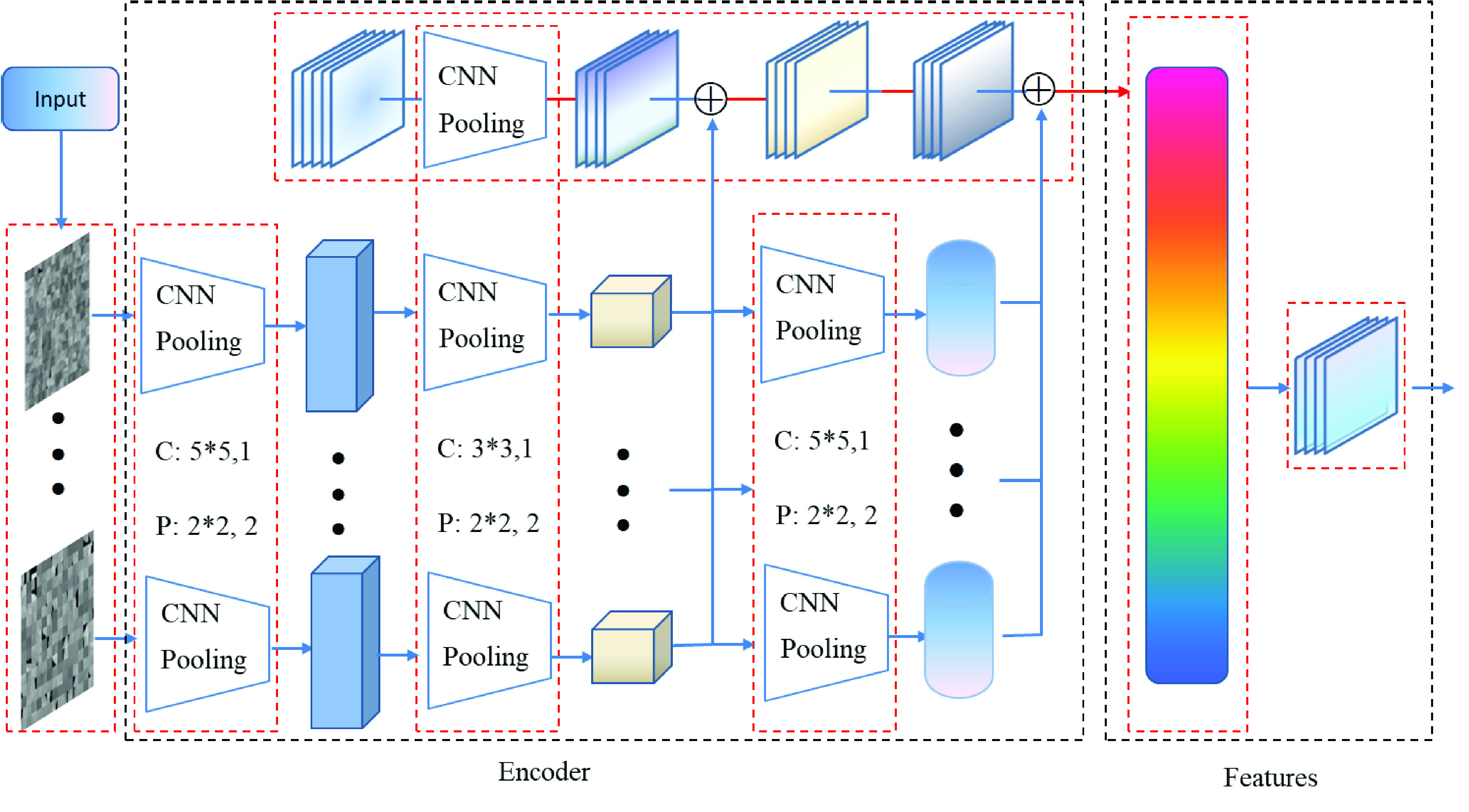
The architecture of feature learning.

Inspired by auto-encoders [30] and GailSet [31], we aim to extend the deep residual network for feature learning and extraction. As the deep residual network had the advantage of strong robustness and feature representation, the residual architecture keeps gradient convergence. The resnet-50 was select for feature learning in this study, the labeled lung regrouped images were as input into resnet-50 network to training a classifier model. The architecture of residual based feature learning can be depicted in FIGURE 8.

#### 1) Residual Encoder Block

Different from auto-encoder, the residual encoder block based feature learning was a classifier. The measure of the quality of feature learning is the accuracy of classifier. While the training process was completed, the deep residual feature learning block with strong feature representation had been built. The input $X$ into the residual encoder block for feature learning, the features $H_{k} $ was extracted from $K^{th}$ layer in (9).

\begin{equation*} H_{k} =f(X\Delta W_{k} +b_{k})\tag{9}\end{equation*}

where denotes the convolution operation, $b_{k} $ denotes residual concatenate. The decoder process can be shown in (10).

\begin{equation*} Z=f^{\prime }(H_{k})\tag{10}\end{equation*}

where $Z$ denotes the output of the decoder block, $f^{\prime }$ denotes the loss function.

#### 2) Pooling

The goal of pooling is to condense a set of lung image information, and was fed to the classification block. To balance the representativeness and the computational cost, the Pooling operate can be demonstrated as follows.

\begin{align*}&\hspace {-.5pc} P=conv(1\times 1)(cat(\max ~pooling, \\& \qquad\qquad\qquad\qquad  meanpooling,averagepooling))\tag{11}\end{align*}

where $P$ means the pooling operate, $cat$ means concatenating on the channel dimension, $conv(1\times 1)$ means $1\times 1$ convolutional layer, and *maxpooling*, *meanpooling* and *averagepooling* are applied to the set dimension.

#### 3) Attention

**FIGURE 9.**
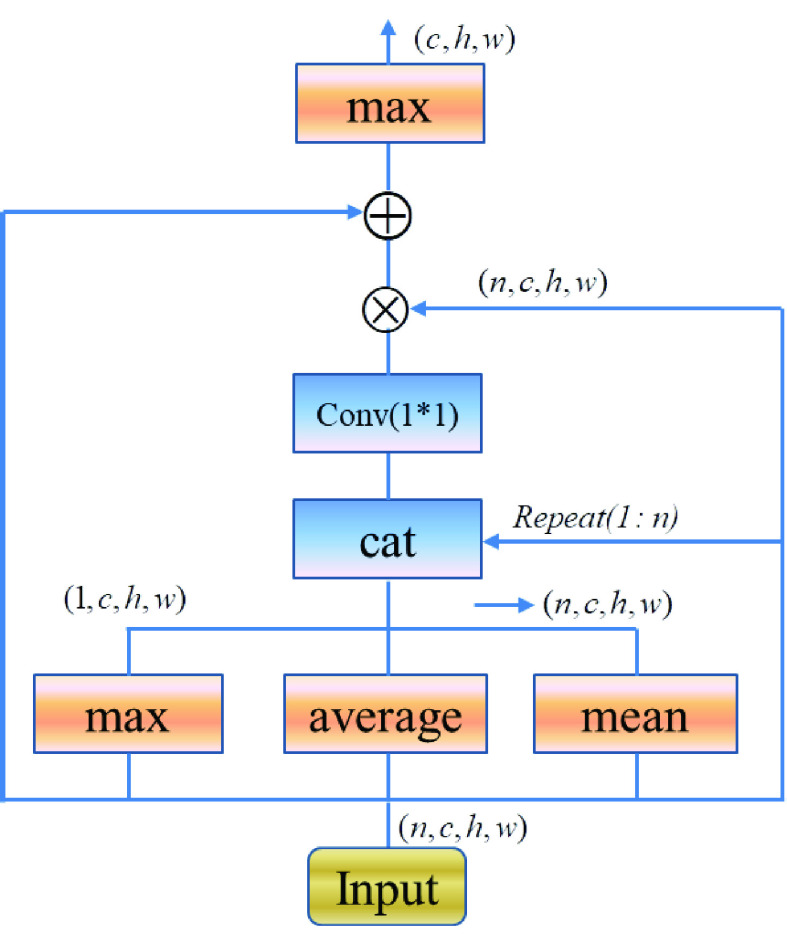
Visual attention was used in this study.

Visual attention is one of the most important mechanisms in human visual system, in computer vision tasks; attention has been successfully applied [32], [33], [34]. In this study, the attention was capitalized to implement *Pooling* operate. As shown in FIGURE 9, the pixel wise attention strategy was introduced, specifically, the output of *Pooling* were refined utilizing the global information to learn an element wise attention map for each image feature map. The global information was first collected by statistical functions, and then was fed into a 1*1 convolutional layer with the original feature map to calculate an attention map for refinement. Here, $n$ represents the number of feature maps in a set, and $c$, $h$ and $w$ denote the number of channels, the height and width of a feature map respectively. The residual block was used to accelerate and stabilize the convergence, which called residual encoder block.

### C. Classification Block

The classification block aims to classify the chest X-ray images according to the extracted features from the residual encoder block. We adopt the Support Vector Machine which has the strong feature representation ability as the backbone of our classification algorithm. Since the SVM had proved as a power tool in machine leaning and data mining with the strong robustness and classification ability [35].

In practice, the high sensitivity and efficient detect COVID-19 cases is the most critical task. For this reason, the labels were divided into two classes: COVID-19 infection cases and others, we concentrated on more feasible work such as distinguishing the confirmed cases in order to take relevant measures as soon as possible to prevent the spread of the epidemic.

The pre-processed lung regrouped images were first masked from the residual encoder block, which were then fed into the SVM classifier for training with the Radial Basis Function kernel function in this study.

## IV. Experiment and Analysis

To verify the effectiveness of the method, the training environment, training and test data and training details were discussed in this section.

### A. Experimental Environment

The high performance computing platform of Northeast Agricultural University is available for training in this study, the details of computing resources are depicted in TABLE 1.

**TABLE 1:** Computer Resources

Hardware and Software	Specifications
OS	Cent OS 7.5
CPU	8* Intel(R) Xeon(R) Silver 4216 CPU @ 2.10GHz
Language	Python 3.8
GPU	NVIDIA GeForce RTX 2080 Ti (11G)
RAM	2*16G
Disk	320G
Framework	Tensorflow2.3.1, Cuda10.1

### B. Data Processing

**FIGURE 10.**
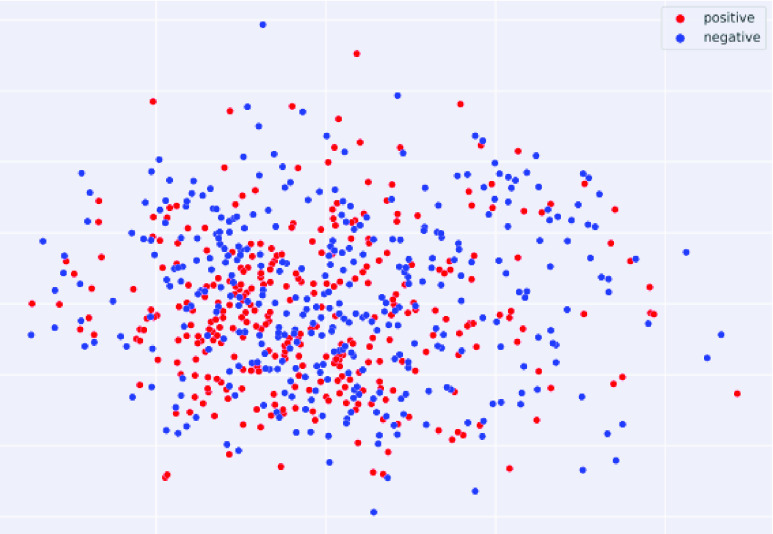
The distribution of the original CXR images.

The public chest X-ray dataset [36] was used in this study, which contains 315 COVID-19 images (positive) and 357 non- COVID-19 images (negative). The distribution of the original CXR images can be depicted in FIGURE 10. As shown in FIGURE 10, the positive and negative samples were hardly to distinguish by unsupervised learning method.

All of the original CXR images were input into the proposed method for pretreatment according algorithm I. When the labeled lung regrouped images were generated, they were split into two parts, 70% for training and 30% for test set.

### C. Training Details

The hyperparameters of deep residual auto-encoder and SVM are shown in TABLE 2.

**TABLE 2:** The Hyperparameters of Proposed Method

Parameter	Value
Input_size	512*512*3
Batch_size	16
Loss function	binary_crossentropy
Activation function	Sigmoid, ReLU
Optimizer function	RMSProp
Epochs	200
Kernel function of SVM	Radial Basis Function

#### 1) Loss Function

The loss function was used for evaluating the gap of input and output, in this paper, the *Binary_crossentropy* function played an important role to penalize the dissimilarity $L$ and $X$ as shown in (12).

\begin{align*}&\hspace {-.5pc} LOSS=-\frac {1}{{\textit {output size}}}\sum \limits _{i=1}^{{\textit {output size}}} {y_{i}} \cdot \log \hat {y_{i}} +(1-y_{i}) \\& \qquad\qquad\qquad\qquad\qquad\qquad\qquad\qquad\quad \cdot \,\log (1-\hat {y_{i}})\tag{12}\end{align*}

#### 2) Activation Function

The activation function in this work adopted sigmoid function and *ReLU* function, which can be shown in (13) and (14) respectively.

\begin{align*} Sigmoid(t)=&\frac {1}{1+\exp ^{-t}} \tag{13}\\ ReLU(t)=&{{\begin{cases} {t,} &\quad {if~t>0} \\ {0,} &\quad {if~t\le 0} \\ \end{cases}}}\tag{14}\end{align*}

#### 3) Optimizer Function

The *RMSProp* optimizer function in this work was used for automatically adjust the learning rate and select different learning rates for each parameter. For each parameter $w^{j}$, the value of $v_{t} $ can be express in (15).

\begin{equation*} v_{t} =\rho v_{t-1} +(1-\rho)\cdot g_{t}^{2}\tag{15}\end{equation*}

where $g_{t} $ denotes the projection of updating parameters and $v_{t}$ denotes the exponential average of squares gradients. The stride can de described in (16).

\begin{equation*} w_{t} =\frac {\tau }{\sqrt {v_{t} +\varepsilon }}\cdot g_{t}\tag{16}\end{equation*}

where $\tau $ denotes the initial learning rate and $\varepsilon $ is a constant $e^{-10}$.

#### 4) Kernel Function

The kernel function in SVM was to map the gap from low to high dimension, in this paper, the radial basis function (RBF) was adopt as shown in (17).

\begin{equation*} K(x,x_{i})=\exp \left({-\frac {\left \|{ {x-x_{i}} }\right \|^{2}}{2\rho ^{2}}}\right)\tag{17}\end{equation*}

### D. Result and Discussion

#### 1) Result Evaluation Index

In order to evaluate the effectiveness of the model more comprehensively, it is necessary to select reasonable evaluation index. In this study, the help of different parameters such as precision, sensitivity, F1 score [37] and accuracy were selected to evaluate the proposed model and the state-of-art models, The calculation methods of different parameters are shown in (18)~(21).

\begin{align*} Precision=&\frac {True ~Positive}{True ~Positive+ False ~Positive} \tag{18}\\ Sensitivity(Recall)=&\frac {True ~Positive}{True~Positive+False~Negative} \qquad ~\tag{19}\\ F1~Score=&2\times \frac {Precision\times Recall}{Precision+Recall} \tag{20}\\ Accuracy=&\frac {True~Positive+True~Negative}{N}\tag{21}\end{align*}

#### 2) Ablation Experiment

To validate the impact of the basic components in the proposed method, an ablation experiment is necessary. TABLE 3 shows the thorough results of ablation experiments on test set, the impact of the basic every component in Section III is studied.

**TABLE 3:** Ablation Experiment

Set	Methods	Accuracy
Original CXR images	ResNet-50	45%
Regrouped lung images	ResNet-50	48%
Original CXR images	ResNet-SVM	85%
Regrouped lung images	ResNet-SVM	93%

In binary classification problem, when under the limited training data, the residual network can hardly achieve a wonderful performance. To address this problem, we combined SVM with the strong robustness and feature representation ability. Experimental results showed that the proposed method can effectively improve the detection accuracy.

#### 3) Compare With Existing Models

Some state-of-art classification models were selected and the recently published COVID-19 detection method for comparison. TABLE 4 shows the classic models and patch-based CNN [6], DarkNet and YOLO [7], COVID-SDNet [17], Hussain *et al.* [4], Novitasari *et al.* [38], Misra *et al.* [39], Jain *et al.* [40], Asif *et al.* [41], Mohammad *et al.* [42], and Apostolopoulos *et al.* [43] proposed method.

**TABLE 4:** Comparison With Existing Models

Set	Precision	Sensitivity	F1 Score	Accuracy
Original CXR images	0.505	0.429	0.46	0.45
Regrouped lung images	0.51	0.45	0.48	0.48
Original CXR images	0.88	0.82	0.85	0.85
AlexNet-SVM	0.736	0.722	0.729	0.74
DenseNet-SVM	0.90	0.76	0.826	0.8219
VGG-SVM	0.852	0.743	0.794	0.7952
PCA-SVM	0.87	0.76	0.81	0.81
Patch-based CNN [6]	0.947	0.90	0.923	0.925
DarkNet and YOLO [7]	0.905	0.835	0.867	0.8702
COVID-SDNet [17]	0.853	0.771	0.81	0.81
Hussain et al. [4]	0.821	0.765	0.792	0.7952
Novitasari et al. [38]	0.96	0.86	0.92	0.9174
Misra, S. et al [39]	0.896	1	0.91	0.933
Jain, R. et al [40]	0.91	0.78	0.86	0.93
Asif Iqbal, et al. [41]	0.90	0.892	–	0.896
Mohammad, et al. [42]	0.85	0.80	–	0.914
Apostolopoulos, et al. [43]	0.933	0.9285	–	0.934
Our method	1	0.88	0.936	0.936

It is not feasible to compare the performance between the proposed method and the state-of-art classification models; the main reason is that there were different data source and number of test samples. However, the performance in terms of precision, sensitivity, F1 score and accuracy along with the different number of images which used for exiting models, the proposed method and ablation terms were summarized in TABLE 4. From this table, the proposed method and Misra *et al.* [39], Jain *et al.* [40], and Apostolopoulos *et al.* [43] had achieved a satisfactory results, but without the proposed method, Misra *et al.* [39] used 3759 training images include healthy images COVID-19 images, Jain *et al.* [40] used 6432 training images, Apostolopoulos *et al.* [43] used 1428 training images, however, the proposed method had training the proposed method with 470 training images and 202 image for test including normal and COVID-19 terms, that was enough prove it can achieve a better performance with limited training samples.

#### 4) Discussion

The proposed method combined machine learning and image processing, which took advantage of the deep residual network and Support Vector Machine algorithms. The prior knowledge in COVID-19 detection is also contributed to this work, we found that the random localized features distributed in lung region, therefore, the image regrouping method was adopted to enhance features and weakened noises interference and the residual auto encoder block was built for feature extraction, and fed them into SVM for classification. Experiment result showed that the proposed method can achieve an expected result.

We aimed to come up with one way of rapid tests, the RT-PCR was widely used in COVID-19 detection, however the low sensitivity may result in false negative and delays the best time for treatment, what’s more terrible is that the highly contagious of COVID-19 may cause people who all direct or indirect contacts are isolated. The CT with a high sensitivity to detect COVID-19 may be a possible method, but the CT process takes a major expenditure of time and effort, which makes the burden of the CT staff even more heavy, the inefficient approach, is not suitable for COVID-19 detection either.

With the unremitting efforts of researchers, the detection method based on machine learning is being accepted by people. More and more detection models provide various solutions for COVID-19 detection have been emerged. The proposed method is one of the methods that had proved to be effective. But this is only the result of a specific experimental environment and has not yet been carried out for clinical validation. We deeply hope that our proposed method can provide meager strength for overcoming epidemic situation.

## V. Conclusion

In this study, we proposed a novel chest X-ray images based COVID-19 detection method, the image regrouping and the residual encoder block combined for feature learning and extraction, the extracted features were input into SVM for classification. The proposed method made a concerted effort to reduce the interference of background and skeleton noise, which showed strong feature learning and expression ability by combined between deep residual network and Support Vector Machine.

In practice, to prevent the rapid spread of the epidemic, a high sensitivity and efficient method for COVID-19 detection is necessary. The proposed method investigated the potential biomarkers in the CXR images and found the random localized distributed in lung region and the noise such as background, spine and ribs have interference of classifiers. To address this problem, we divided the lung region into $15\times 15px$ pieces, and randomly regrouped them in to a new image; the regrouped image kept the original COVID-19 features and weakened the background and ribs noises.

We also proposed a residual encoder block for feature learning, which inspired from auto-encoder. The purposed of auto-encoder is learning a strong feature for image reconstruction; rather, we concentrated on more feasible work such as feature classification to detection COVID-19. Since the deep residual network had achieved the state-of-art in classification but need large-scale training data. As this reason, we extend the residual network for feature extraction and input the extracted features into SVM for classification. Experiment results showed that, the proposed method outperformed the existing classification models in COVID-19 detection.

Based on the above work, we hope that our work will contribute to fight against the epidemic around the world. Pray for the early elimination of the epidemic.
